# Differential seasonal performance of C3-epi-D3 level and proportion on multiple metabolic disorders in patients with type 2 diabetes mellitus

**DOI:** 10.1186/s40001-024-02212-9

**Published:** 2024-12-23

**Authors:** Xiaohong Chen, Bi Peng, Wenchun Ye, Bitao Wu, Qiang Yang, Jie Tang, Yuwei Yang

**Affiliations:** 1 Mianyang Central Hospital, Affiliated to School of Medicine, University of Electronic Science and Technology of China, Mianyang, China; 2 The Third Hospital of Mianyang, Sichuan Mental Health Center, Mianyang, China

**Keywords:** Type 2 diabetes mellitus, Metabolic disorders, C3-epimer, 25 hydroxyvitamin D3, Association

## Abstract

**Background and aim:**

Recent Mendelian randomization and meta analysis suggest a controversial causality between C3-epimer of 25 hydroxyvitamin D3 (C3-epi-D3) and type 2 diabetes mellitus (T2DM). The clinical evidence regarding the impact of C3-epi-D3 on the progression of T2DM is currently insufficient. This study aims to investigate whether C3-epi-D3 has any effect on metabolic disorders of T2DM patients.

**Methods:**

A total of 1222 patients with T2DM were prospectively enrolled in this study and followed up every 1 to 2 months for 3 to 6 months. Kidney biomarkers, lipids, electrolytes, and 25 hydroxyvitamin D (25-OHD) metabolites were measured as required during follow-up, to investigate the association of C3-epi-D3 levels and %C3-epi-D3 with metabolic disorders, including dyslipidemia, chronic kidney disease (CKD), and calcium-phosphorus metabolic disorder.

**Results:**

Among these T2DM patients, there were age and seasonal differences in C3-epi-D3 levels (*χ*^2^ = 10.419 and 19.609, *P* = 0.034 and < 0.001), but only seasonal difference in %C3-epi-D3 (*χ*^2^ = 79.299, *P* < 0.001). C3-epi-D3 levels showed an evident correlation with calcium-phosphorus product during autumn and winter (*ρ* = − 0.336 and − 0.304, both *P* < 0.001), and was confirmed as an independent factor on calcium-phosphorus metabolic disorder during autumn and winter by subsequent partial correlation analysis (*r*_*partial*_ = − 0.300 and − 0.319, both *P* < 0.001). Both C3-epi-D3 levels and %C3-epi-D3 showed evident correlation with the severity of chronic kidney disease (CKD) in summer (*ρ* = 0.344 and 0.445, both *P* < 0.001). But subsequent multinomial logistic regression confirmed that only %C3-epi-D3 independently associated with moderate CKD severity in summer (*OR* = 1.348, *P* < 0.001), as well as serious CKD severity in spring, summer, and autumn (*OR* = 1.324, 1.342, and 1.698, all *P* < 0.001). Additionally, no evident correlation was observed between C3-epi-D3 and dyslipidemia.

**Conclusion:**

Our study releases a seasonally differential impact of C3-epi-D3 levels and proportions on metabolic disorders of T2DM patients, considering to be potentially related to their pathogenesis of different metabolic disorders. The independent association between %C3-epi-D3 and CKD suggests a potential pathological relevance involving C3-epi-D3 itself.

**Supplementary Information:**

The online version contains supplementary material available at 10.1186/s40001-024-02212-9.

## Introduction

Type 2 diabetes mellitus (T2DM) is the most common type of diabetes mellitus, accounts for nearly 90% of the diabetes cases worldwide (approximately 480 million) [1]. It is a metabolic disease caused by multiple etiologies and characterized by chronic hyperglycemia. Its etiology of hyperglycemia can be distilled to insulin resistance (i.e., progressive impairments in insulin sensitivity) and islet failure (i.e., insulin relative hyposecretion of pancreatic islets lead to a loss of compensation for the decline in insulin sensitivity) [2]. Due to the extensive metabolic regulatory impact of insulin, T2DM patients usually present a range of metabolic disorders, including aberrant metabolism of glucose, lipid, protein, and electrolyte, etc. Among these metabolic disorders, dyslipidemias with the prevalence as high as 75% contributes significantly to the elevated risk of cardiovascular disease including chronic kidney disease (CKD) [3]. Additionally, dysnatremia and dyskalemia are associated with diabetic ketoacidosis and hyperosmolar hyperglycemic state [4]. Aberrant protein metabolism is very complex in T2DM. The hyperglycemic cell can change protein synthesis, secretion, and catabolism through chemical modification caused by metabolic intermediates [5, 6]. Therefore, proactive management of metabolic disorders is imperative for T2DM patients, especially for prevention and mitigation of dyslipidemias and subsequent cardiovascular complications.

The proactive management of metabolic disorders involves various dimensions in T2DM patients. T2DM patients usually exhibit a notable deficiency in vitamin levels, and vitamin supplementation emerges as an alternative therapeutic strategy. The insufficiency of antioxidant vitamins (A, C, E) and B vitamins is considered in relation to associated with excessive oxidative stress induced by glucose control [7, 8] and some degree of metabolic dysregulation [9–11], respectively. The deficiency of vitamin D is considered as a risk for DM event and its complications, particularly cardiovascular complications [12–14]. In contrast, vitamin D deficiency is even more harmful for patients with T2DM. The primary physiological function of vitamin D is to regulate calcium and phosphorus metabolism. Recent systematic reviews and meta-analyses have indicated a close association between vitamin D deficiency and the occurrence of diabetic kidney disease [15] and dyslipidemia [16]. These evidences suggests that vitamin D plays a crucial role in multiple metabolic disorders, including electrolyte and lipid metabolic disturbances, even the development of chronic kidney disease (CKD).

Recently, C3-epimers of 25-hydroxyvitamin D (C3-epi-D) have sparked a hot topic on the field of vitamin D nutrition. The phenomenon of C3-epimerization has long been observed during the chemical synthesis of vitamin D analogs [17]. But until 2006, C3-epi-D were first detected in human peripheral blood using a high-performance liquid chromatography-tandem mass spectrometry (HPLC–MS/MS) [18]. The spatial isomer C3-epi-D is mainly generated from the C3-epimerization of natural 25-hydroxyvitamin D (25-OHD), but has negligible or no bioactivity [19] due to β → α epimerization at the C3 position [20]. The elevated C3-epi-D level has been consistently observed in various conditions, including DM [21, 22], which may potentially lead to an overestimation of 25-OHD storage [23] and subsequent clinical risks [24]. In a recent study conducted on the EPIC-InterAct cohort, meta analysis found a positive association between C3-epi-D3 and T2DM, but Mendelian randomization analysis ruled out the causality between them [25]. To date, little is revealed about the pathological relevance of C3-epi-D with T2DM. This study aims to investigate the association of C3-epi-D levels and proportions with metabolic disorder of T2DM patients, to provide evidence on the pathological relevance of C3-epi-D during T2DM progression.

## Methods

### Patients

From Jan. 2019 to Dec. 2022, a total of 1222 T2DM patients were continuously enrolled from Endocrinology Clinic of Mianyang Central Hospital, affiliated to School of Medicine, University of Electronic Science and Technology of China. All patients were followed up every 1 to 2 months for 3 to 6 months. At each follow-up visit, their kidney biomarkers were measured as needed. At the third follow-up visit, their lipids, electrolytes, and 25-OHD metabolites were measured as needed.

Inclusion criteria: (1) Patients were diagnosed as T2DM according to the guideless of American Diabetes Association (ADA) [26], with fasting glucose ⩾ 7.0 mmol/L or random glucose (or postprandial 2 h-glucose) ⩾ 11.1 mmol/L or hemoglobin A1c ⩾ 6.5%; (2) Patients were regularly followed up at the Endocrinology Clinic every 1 to 2 months for a duration of 3 to 6 months, during which their urinary albumin/creatinine ratio (UACR), serum creatinine (SCr) and cystatin C (CysC) levels were measured; (3) Patients expressed willingness to provide blood and urine samples for kidney function, electrolytes, lipids, and vitamin D analysis at the third follow-up.

Exclusion criteria: (1) Patients with a diagnosis of type 1 diabetes mellitus or secondary diabetes mellitus; (2) Pregnant women; (3) Patients with comorbidities that significantly interfere with the assessment of vitamin D levels and CKD severity, such as severe pulmonary insufficiency, primary cardiac diseases, primary nephropathy, severe infection, autoimmune diseases, thyroid disorders, and malignant tumors; (4) Patients who have been taking vitamin D supplementation or any medication other than T2DM drugs within the past month.

### Sample collecting and processing

Aliquots of 3.0 ml fasting blood were collected in each of two SST-II vacuum tubes (BD, USA) during the third follow-up, and centrifuged at approximately 1500×*g* for 10 min to separate serum. One was used for measuring kidney biomarkers, electrolytes, and lipids within 2 h. Another was used for measuring the levels of 25-OHD metabolites within 3 d. If not immediately analyzed, serum was stored the at 2–8 °C for less than 7d [27]. After the collection of blood samples, patients were instructed to reserve approximately 3 ml of random urine for measuring UACR within 2 h.

Before the detection of 25-OHD metabolites, serum was subjected to preprocessing to obtain supernatant using a lipid-soluble vitamin assay kit (CatNo.1001040, FIND Biotech, CHN). A total of 200 μl serum was mixed with 10 μl of a mixture internal standard consisting of 25-OHD2-d6/25-OHD3-d6/C3-epi-D3-2H3, and vortexed with 1.0 ml tertbutyl-methyl-ether releaser for 5 min, followed by centrifugation at 12,000×*g* for another 5 min. Took 800 μl supernatant to dry using an MD200-1A Nitrogen Evaporator (Allsheng, China), and redissolved the desiccative substance in 100 μl methanol solution containing 0.1% formic acid. After vortex mixing for 2 min and centrifugation at 12,000×*g* for 5 min, only 50 μl resulting redissolution supernatant was absorbed into a sample microplate and sealed for chromatographic analysis.

### Determination of kidney biomarkers, electrolytes, and lipids

Serum and urinary albumin were measured with the bromocresol green method and with the modified immunoturbidimetric method, respectively; serum and urinary creatinine were both measured with the sarcosine oxidase method; serum total-protein was measured with the biuret method; serum urea was measured with the urease-glutamate dehydrogenase method; serum total cholesterol (total-Chol) was measured with the cholesterol oxidase method; serum triglyceride was measured with the glycerol phosphate oxidase method; serum CysC, high-density lipoprotein cholesterol (HDL-Chol), and low-density lipoprotein cholesterol (LDL-Chol) were measured with the immunoturbidimetric method; serum total calcium was measured with the *o*-crephenolphthalein complexometric ketone method; serum inorganic phosphorus was measured with the molybdenum blue method. All the above kits for serum and urinary biomarker assay were provided by Sichuan Maccura Biotechnology Co., Ltd. and Chongqing BioStec Biotechnology Co., Ltd., respectively. And all the above serum and urinary biomarkers were measured on the LST008 autoanalyzer (HITACHI, Japan) and the A25 automatic special protein analyzer (BioSystems, Spain), respectively.

The dyslipidemias were defined as meeting with any of the following items: total-Chol ⩾ 5.2 mmol/l, triglyceride ⩾ 1.7 mmol/l, HDL-Chol < 1.0 mmol/l, LDL-Chol ⩾ 3.4 mmol/l [28]. The eGFR was calculated using CKD-EPI CysC-Cr formula [29]: eGFR = 135 × min(SCr/κ,1)^α^ × max(Cr/κ,1)^−0.601^ × min(CysC/0.8,1)^−0.375^ × max(CysC/0.8,1)^−0.711^ × 0.995^Age^ × 0.969 (if female), where *κ* = 62 μmol/L(female)/80 μmol/l(male), *α* =  − 0.248 (female)/− 0.207 (male). The UACR was calculated using the following formula: UACR (mg/g) = urinary albumin (mg/l)/urinary creatinine (g/l). The calcium-phosphorus product (Ca-IP) was defined as the product of total calcium and inorganic phosphorus measured in mg/dl unit, which is often used to evaluate the calcium-phosphorus metabolism. So, Ca-IP [(mg/dl)^2^] = [4 × total calcium (measured as mmol/l)] × [3.1 × inorganic phosphorus (measured as mmol/l)].

### Chromatography tandem mass spectrometry assay of 25-OHD metabolites

25-OHD metabolites were measured on a Jasper™ HPLC–MS/MS (Shimadzu, Japan)/AB SCIEX™ 4500MD triple quadrupole mass spectrometer (ABI, USA). UHPLC/MS conditions had been reported in our previous study [30]. Via monitoring the Q1/Q3 mass = 383:3 Da/365:3 Da ion pairs, C3-epi-D3 and 25-OHD3 were distinctly separated and identified with the retention time of 3.48 min and 3.40 min, respectively (Supplementary Fig. 1).

Internal standard detection scheme: 25-OHD2-D6, 25-OHD3-D6 and C3-epi-D3-2H3 were used as internal standards for 25-OHD2, 25-OHD3 and C3-epi-D3 to calculate the peak area ratio, respectively. Five gradient concentration standards were utilized to draw the “peak area ratio-concentration” curve for each 25-OHD metabolite, which was used to quantify the concentration in sample. %C3-epi-D3 was defined as the C3-epi-D3 concentration divided by the sum of the C3-epi-D3 and 25-OHD3 concentrations.

### Statistical analysis

Statistical analysis was performed in the MedCalc software v20.1 (MedCalc, Belgium) and SPSS software v22.0 (SPSS, USA). The measurement data were presented as *median* (*P*_*25*_, *P*_*75*_) [*min*, *max*]. The missing values were addressed using the case elimination method. Two- or multiple-group difference was analyzed using Mann–Whitney U or Kruskal–Wallis test. Pairwise comparison among multiple groups was performed by the POST-HOC comparisons, and the adjusted *P* value (*Padj*) was employed to determine the significance. The Spearman correlation coefficient (*ρ*) was analyzed between 25-OHD3 metabolite and each metabolic disorder, the significant correlation coefficient |*ρ*|< 0.1, 0.1 ≤|*ρ*|< 0.3, 0.3 ≤|*ρ*|< 0.5, 0.5 ≤|*ρ*|< 0.7, 0.7 ≤|*ρ*|< 0.9, and |*ρ*|≥ 0.9 indicate no, weak, mild, moderate, strong, and extremely strong correlation, respectively. If |*ρ*|≥ 0.3 were considered as evident correlation [31], furtherly displayed using a line-heatmap. The line visually displays the magnitude of Spearman correlation coefficient. The heatmap visually displays the distribution of observation points via color coding of gradual decreasing density from red to orange, green, blue, and finally white. The partial correlation analysis and multinomial logistic regression were used to verify the association of Ca-IP and CKD severity with 25-OHD metabolites after adjusting for confounders, respectively.

## Results

### Clinical information of the patients with T2DM

This study enrolled 1222 patients with T2DM, and no gender difference was observed in their general information and chronic history (all *P* > 0.05).

### Age, gender, and seasonal differences in 25-OHD metabolite levels

**Fig. 1:**
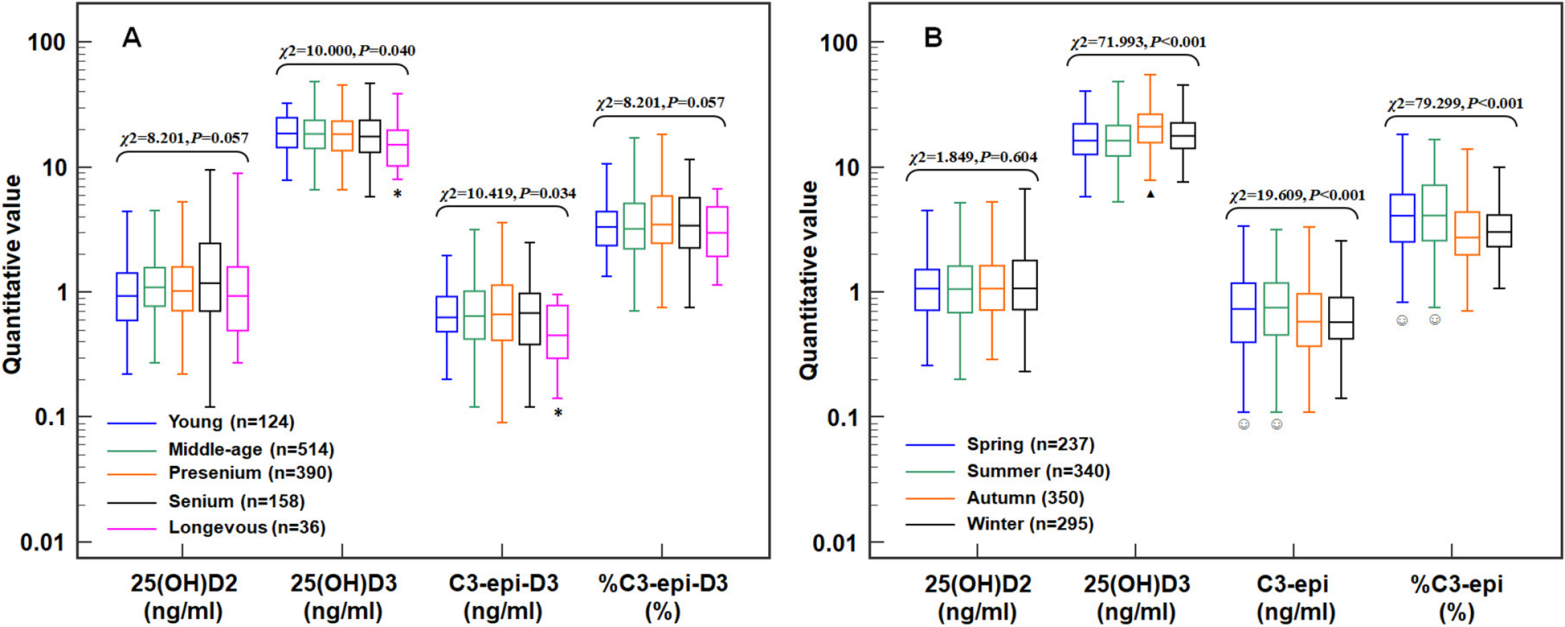
Age and seasonal differences in 25-OHD metabolite levels in patients with DM. **A** Box-and-whisker plot of age groups, * vs. the other age groups, *Padj* < 0.05; **B** Box-and-whisker plot of seasonal groups, ^▲^ vs. the other seasons, *Padj* < 0.05; ^☺^ vs. autumn and winter, *Padj* < 0.05. Results indicated that in patients with T2DM, C3-epi-D3 levels decreased during the longevous period and increased during spring and summer, while %C3-epi-D3 increased during spring and summer

According to recommendation of 2021 WHO age classification [32], all the participants were divided into 5 age groups: young period (*n* = 124, less than 45 years old), middle-age period (*n* = 514, 45 to less than 60 years old), presenium period (*n* = 390, 60 to less than 75 years old), senium period (*n* = 158, 75 to less than 90 years old) and longevous period (*n* = 36, over 90 years old). The group settings for gender and season were as follows: male (*n* = 735) and female (*n* = 487); spring (*n* = 237, Mar. to May), summer (*n* = 340, Jun. to Aug.), autumn (*n* = 350, Sep. to Nov.), and winter (*n* = 295, Dec. to Feb.). By the Kruskal–Wallis test, significant differences were observed among age groups for both 25-OHD3 (*χ*^2^ = 10.000, *P* = 0.040) and C3-epi-D3 (*χ*^2^ = 10.419, *P* = 0.034) (shown in Fig. 1A), also were observed among season groups for 25-OHD3 (*χ*^2^ = 71.993, *P* < 0.001), C3-epi-D3 (*χ*^2^ = 19.609, *P* < 0.001) and %C3-epi-D3 (*χ*^2^ = 79.299, *P* < 0.001) (shown in Fig. 1B). Further POST-HOC analysis showed that the 25-OHD3 and C3-epi-D3 levels were lower in the longevous period compared to the previous four periods (all *Padj* < 0.05), and 25-OHD3 was higher in autumn compared to the other seasons (all *Padj* < 0.05), while C3-epi-D3 and %C3-epi-D3 levels were higher in spring and summer compared to autumn and winter (all *Padj* < 0.05). By the Mann–Whitney test, the gender differences were observed in 25-OHD2 (*z* = − 2.432, *P* = 0.015) and 25-OHD3 (*z* = 4.631, *P* < 0.001), but not in C3-epi-D3 (*z* = 0.631, *P* = 0.528) and %C3-epi-D3 (*z* = −1.798, *P* = 0.072).

### Biomarker levels in DM patients with different CKD severity groups

According to the classification criteria of CKD severity [33] (as detailed in Table 1), 1222 DM patients were categorized into three groups: mild, moderate, and serious CKD groups. By the POST-HOC analysis (shown in Table 2), age, 25-OHD3, %C3-epi-D3, total protein, albumin, urea, SCr, CysC, eGFR, and UACR had significant differences in both moderate and serious CKD groups compared to the mild CKD group (all *Padj* < 0.05). Among these biomarkers, only %C3-epi-D3, albumin, urea, SCr, CysC, and eGFR had significant differences between the moderate CKD group and the serious CKD group (all *Padj* < 0.05). Except for the above biomarkers, in comparison with the mild CKD group, triglyceride had also significant differences in the moderate CKD group (*Padj* < 0.05), while C3-epi-D3 and inorganic phosphorus had also significant differences in the serious CKD group (both *Padj* < 0.05).

**Table 1:** The general information and chronic disease history of the T2DM patients

	Male (*n* = 735)	Female (*n* = 487)	*t/χ*^2^	*P*
Age (years)	59.9 ± 12.2	61.2 ± 13.6	1.664	0.096
BMI (kg/m^2^)	24.0 ± 9.6	22.9 ± 11.9	− 1.821	0.069
Hypertension [*n* (%)]	272 (37.0)	157 (32.2)	2.921	0.087
Hyperuricemia [*n* (%)]	161 (21.9)	126 (25.9)	2.564	0.109
Dyslipidemia [*n* (%)]	531 (72.2)	335 (68.8)	1.694	0.193
CAD [*n* (%)]	131 (17.8)	100 (20.5)	1.403	0.236
CKD [*n* (%)]	253 (34.4)	194 (39.8)	3.698	0.055

*BMI* body mass index, *CAD* coronary artery disease, *CKD* chronic kidney disease

**Table 2:** Comparison of laboratory results among patients with different severity of CKD

Indicator	Mild CKD		Moderate CKD		Serious CKD		*χ*^2^	*P*
	*n*	M (P25, P75)	n	M (P25, P75)	*n*	M (P25, P75)		
Male/female (*n*/*n*)	775	482/293	204	121/83	243	132/111	4.854	0.088
Age (years)	775	56 (50, 66)	204	64 (55, 73) ^▲^	243	65 (56, 76) ^▲^	92.458	< 0.001
25-OHD2 (ng/ml)	775	1.07 (0.71, 1.65)	204	1.05 (0.75, 1.75)	243	1.05 (0.68, 1.58)	1.601	0.449
25-OHD3 (ng/ml)	775	19.27 (14.58, 24.98)	204	16.14 (13.19, 20.73) ^▲^	243	15.72 (11.24, 21.49) ^▲^	62.173	< 0.001
C3-epi-D3 (ng/ml)	775	0.61 (0.42, 0.93)	204	0.62 (0.36, 0.99)	243	0.86 (0.41, 1.44) ^▲^	24.404	< 0.001
%C3-epi-D3 (%)	775	3.11 (2.24, 4.52)	204	3.36 (2.55, 5.57) ^▲^	243	5.82 (2.40, 9.37) ^▲☺^	72.218	< 0.001
Total protein (g/l)	775	72.6 (68.0, 76.5)	204	70.7 (65.0, 74.5) ^▲^	243	69.0 (63.1, 74.2) ^▲^	39.096	< 0.001
Albumin (g/l)	775	45.4 (42.7, 48.0)	204	43.1 (39.7, 46.3) ^▲^	243	41.8 (37.9, 45.9) ^▲☺^	95.657	< 0.001
Urea (mmol/l)	775	5.83 (4.75, 8.46)	204	7.33 (5.31, 10.12) ^▲^	243	8.55 (6.28, 12.72) ^▲☺^	73.069	< 0.001
SCr (μmol/l)	775	60.7 (42.3, 71.3)	204	58.1 (37.8, 82.5) ^▲^	243	71.0 (51.3, 107.8) ^▲☺^	53.559	< 0.001
CysC (mg/l)	775	0.95 (0.83, 1.08)	204	1.08 (0.89, 1.29) ^▲^	243	1.31 (1.03, 1.70) ^▲☺^	237.729	< 0.001
eGFR (ml/min/1.73m^2^)	775	95.8 (81.5, 116.5)	204	86.2 (61.9, 112.7) ^▲^	243	65.5 (45.1, 93.7) ^▲☺^	159.784	< 0.001
Triglyceride (mmol/l)	775	1.79 (1.20, 3.02)	204	1.63 (1.17, 2.49) ^▲^	243	1.66 (1.22, 2.32)	6.263	0.044
Total-Chol (mmol/l)	775	4.94 (4.14, 5.72)	204	5.06 (4.03, 6.06)	243	4.99 (3.87, 5.90)	0.702	0.704
LDL-Chol (mmol/l)	775	2.92 (2.30, 3.51)	204	2.97 (2.36, 3.97)	243	2.76 (2.07, 3.84)	3.948	0.139
HDL-Chol (mmol/l)	775	1.16 (0.97, 1.44)	204	1.17 (1.00, 1.44)	243	1.21 (1.01, 1.42)	2.151	0.341
Total calcium (mmol/l)	287	2.31 (2.20, 2.41)	153	2.32 (2.19, 2.42)	151	2.26 (2.15, 2.43)	3.829	0.147
Inorganic phosphorus (mmol/l)	287	1.09 (0.98, 1.22)	153	1.10 (1.01, 1.25)	151	1.17 (1.00, 1.29) ^▲^	8.258	0.016
Ca-IP [(mg/dl)^2^]	287	31.3 (27.8, 35.1)	153	31.8 (27.4, 36.7)	151	32.6 (27.8, 37.5)	2.817	0.245
UACR (mg/gCr)	775	9.2 (5.6, 13.8)	204	47.4 (30.5, 128.3) ^▲^	243	224.8 (35.3, 631.0) ^▲^	581.430	< 0.001

*Ca-IP* calcium-phosphorus product. *^▲^ vs. mild CKD, *Padj* < 0.05; ^☺^ vs. moderate CKD, *Padj* < 0.05

### Spearman correlation analysis of 25-OHD3 metabolites with CKD severity, dyslipidemias, and calcium-phosphorus metabolism

**Fig. 2:**
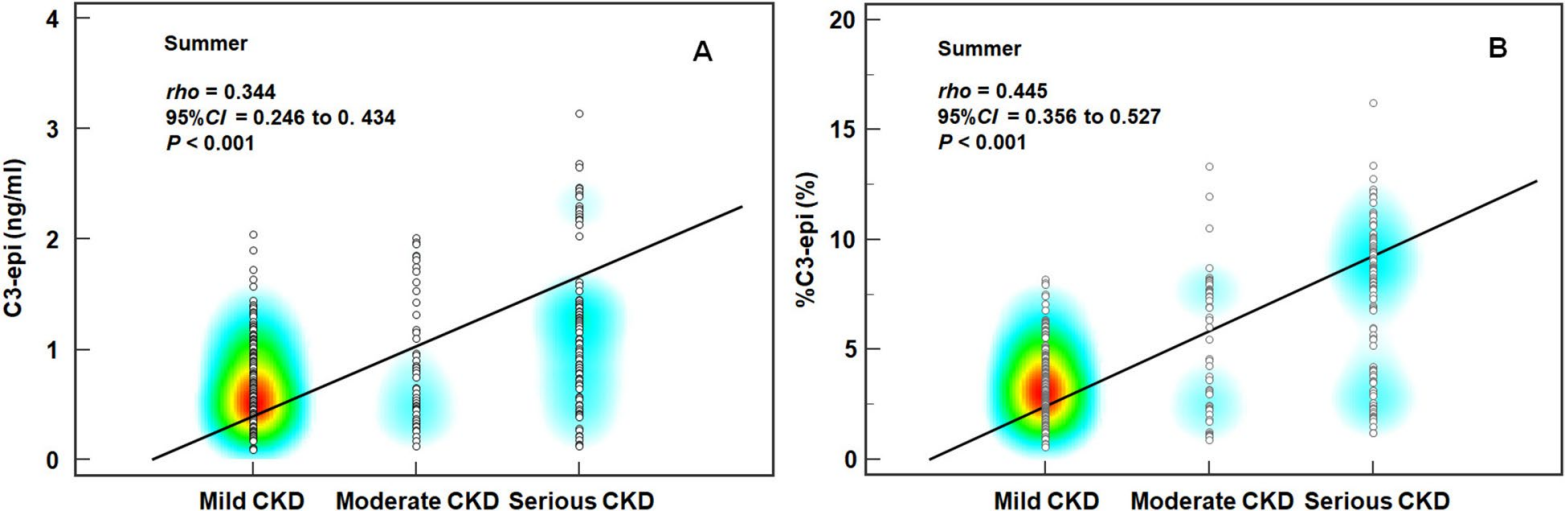
Correlation between C3-epi-D3 levels/proportions and CKD severity. Line-heatmap of Spearman correlation between CKD severity and: **A** C3-epi-D3 levels; **B** %C3-epi-D3. The line and heatmap visually display the magnitude of Spearman correlation coefficient and the distribution of observation points, respectively. Our results suggested an evident correlation between C3-epi-D3 levels/proportions and CKD severity only during summer

**Fig. 3:**
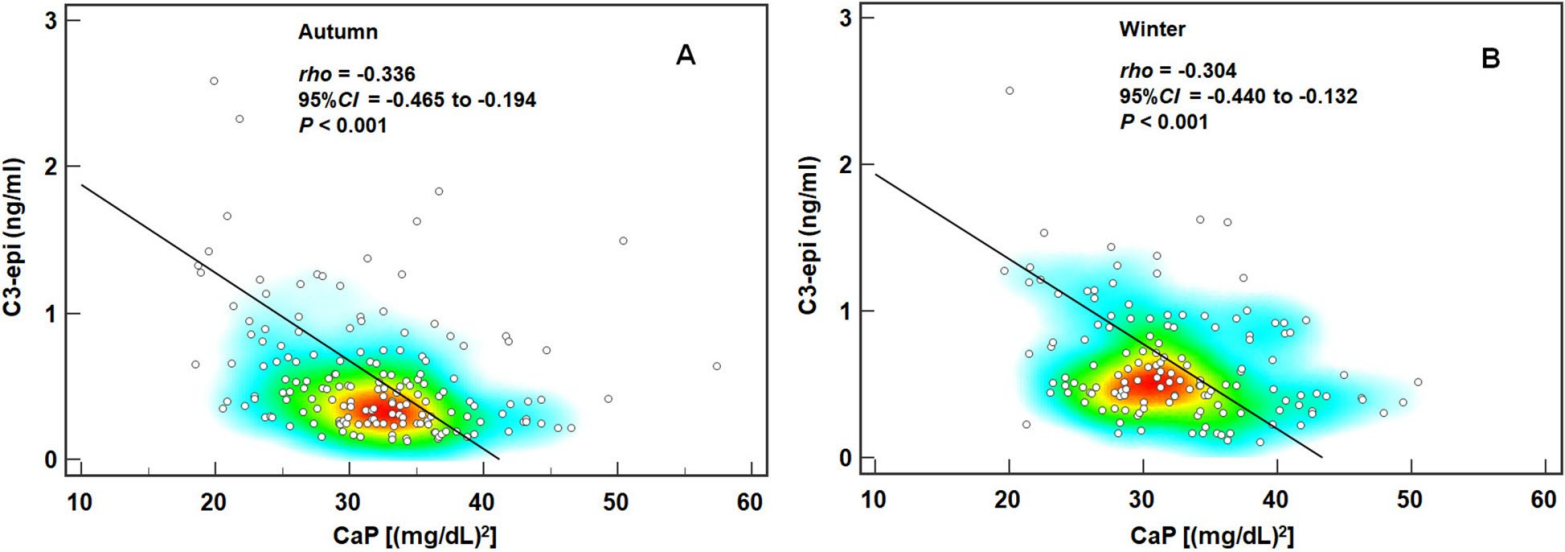
Correlation between C3-epi-D3 levels and Ca-IP in summer. Line-heatmap of Spearman correlation between C3-epi-D3 levels and Ca-IP: **A** during autumn; **B** during winter. The line and heatmap visually display the magnitude of Spearman correlation coefficient and the distribution of observation points, respectively. Our results suggested an evident correlation between C3-epi-D3 levels and Ca-IP during autumn and winter

Using seasonal stratification, we investigated the Spearman correlation between each 25-OHD3 metabolite and CKD severity, or calcium-phosphorus metabolism, or dyslipidemias. It was recognized no correlation when |*ρ*|< 0.1 and evident correlation when |*ρ*|≥ 0.3 [31]. The results presented that only weak correlation, rather than evident correlation, existed between CKD severity and 25-OHD3 level either across whole year or in each season (*ρ* = − 0.226 ~ − 0.151, all *P* < 0.05), also between CKD severity and C3-epi-D3 level (*ρ* = 0.111, *P* < 0.001)/%C3-epi-D3 (*ρ* = 0.230, *P* < 0.001) across whole year (Table 3). But CKD severity was evidently correlated with both C3-epi-D3 (Fig. 2A) and %C3-epi-D3 (Fig. 2B) in summer (*ρ* = 0.344 and 0.445, both *P* < 0.001). As the calcium and phosphorus storage biomarker, Ca-IP was weakly correlated to C3-epi-D3 level across whole year (*ρ* = − 0.161, *P* > 0.05) and %C3-epi-D3 in autumn (ρ = − 0.245, P = 0.001) (Table 3), but evidently correlated to C3-epi-D3 level in autumn (*ρ* = − 0.336, *P* < 0.001) (Fig. 3A) and winter (*ρ* = − 0.304, *P* < 0.001) (Fig. 3B). As for dyslipidemias, it was weakly correlated with 25-OHD3 and C3-epi-D3 only in spring (*ρ* = − 0.166 and − 0.149, *P* = 0.011 and 0.022) (Table 3). These findings suggest a distinct seasonal heterogeneity in the correlations between 25-OHD3 metabolites and the CKD severity, as well as calcium-phosphorus metabolism. However, 25-OHD3 metabolites had little association with dyslipidemias.

**Table 3:** Spearman correlation coefficient between 25-OHD3 metabolites and CKD severity in DM patients

Seasons	25-OHD3 level		C3-epi-D3 level		%C3-epi-D3	
	*ρ* (95%CI)	*P*	*ρ* (95%CI)	*P*	*ρ* (95%CI)	*P*
CKD severity						
All season (*n* = 1222)	− 0.227 (− 0.279, − 0.173)	0.001	0.111 (0.055, 0.166)	< 0.001	0.230 (0.176, 0.283)	< 0.001
Spring (*n* = 237)	− 0.151 (− 0.274, − 0.025)	0.020	0.016 (− 0.112, 0.143)	0.808	0.104 (− 0.024, 0.229)	0.109
Summer (*n* = 340)	− 0.198 (− 0.298, − 0.093)	< 0.001	**0.344 (0.246, 0.434)**	**< 0.001**	**0.445 (0.356, 0.527)**	**< 0.001**
Autumn (*n* = 350)	− 0.226 (− 0.323, − 0.124)	< 0.001	− 0.003 (− 0.107, 0.102)	0.960	0.113 (0.009, 0.216)	0.034
Winter (*n* = 295)	− 0.208 (− 0.315, − 0.096)	< 0.001	− 0.027 (− 0.141, 0.088)	0.647	0.092 (− 0.022, 0.204)	0.114
Ca-IP						
All season (*n* = 591)	− 0.089 (− 0.169, − 0.009)	0.030	− 0.161 (− 0.239, − 0.082)	< 0.001	− 0.098 (− 0.177, − 0.018)	0.017
Spring (*n* = 119)	− 0.048 (− 0.226, 0.133)	0.606	− 0.065 (− 0.242, 0.116)	0.482	− 0.033 (− 0.212, 0.148)	0.719
Summer (*n* = 168)	− 0.046 (− 0.196, 0.106)	0.555	− 0.072 (− 0.221, 0.080)	0.352	− 0.042 (− 0.193, 0.110)	0.585
Autumn (*n* = 166)	− 0.101 (− 0.250, 0.052)	0.194	− **0.336 (**− **0.465, **− **0.194)**	**< 0.001**	− 0.245 (− 0.383, − 0.097)	0.001
Winter (*n* = 138)	− 0.166 (− 0.324, 0.002)	0.052	− **0.304 (**− **0.440, **− **0.132)**	**< 0.001**	− 0.120 (− 0.281, − 0.048)	0.161
Dyslipidemias						
All season (*n* = 1222)	− 0.018 (− 0.074, 0.038)	0.523	− 0.060 (− 0.116, − 0.004)	0.034	− 0.058 (− 0.113, − 0.001)	0.045
Spring (*n* = 237)	− 0.166 (− 0.287, − 0.039)	0.011	− 0.149 (− 0.271, − 0.022)	0.022	− 0.100 (− 0.225, 0.028)	0.124
Summer (*n* = 340)	− 0.009 (− 0.115, 0.098)	0.869	− 0.073 (− 0.178, 0.034)	0.179	− 0.047 (− 0.153, 0.060)	0.387
Autumn (*n* = 350)	0.036 (− 0.069, 0.140)	0.504	0.035 (− 0.070, 0.140)	0.511	− 0.025 (− 0.129, 0.080)	0.641
Winter (*n* = 295)	− 0.030 (− 0.085, 0.143)	0.613	− 0.094 (− 0.206, 0.021)	0.109	− 0.098 (− 0.210, 0.017)	0.094

Based upon |*ρ*|≥ 0.3 considered as evident correlation [30], the results revealed that during specific season, there was a credible correlation between %C3-epi-D3 and CKD severity, as well as between C3-epi-D3 level and Ca-IP. Evident correlations are in bold

### Association degree of 25-OHD3 metabolites with CKD severity and calcium-phosphorus metabolism

Based upon seasonal stratification and adjustment for gender, age, and 25-OHD2 level, we used stepwise multinomial logistic regression and multiple linear regression to analyze the associations between 25-OHD3 metabolites with CKD severity and calcium-phosphorus metabolism, respectively. The multinomial logistic regression analysis revealed significant correlations between 25-OHD3, C3-epi-D3, and %C3-epi-D3 with the progression of moderate and serious CKD across whole year (all *P* < 0.001). However, seasonal stratification analysis showed that only %C3-epi-D3 showed an association with the moderate CKD severity in summer (*OR* = 1.348, P < 0.001), as well as with the serious CKD severity in spring, summer, and autumn (OR = 1.324 ~ 1.698, *P* < 0.001) (Table 4). The multiple linear regression analysis revealed significant association between C3-epi-D3 and the Ca-IP level across whole year (*r*_*partial*_ = − 0.164, *P* < 0.001). However, in seasonal stratification analysis, this association was only remained in autumn (*r*_*partial*_ = − 0.300, *P* < 0.001) and winter (*r*_*partial*_ = − 0.319, *P* < 0.001) (Table 5). These findings suggest that C3-epi-D3 and %C3-epi-D3 levels might have a differential pathological relevance in CKD progression and calcium-phosphorus metabolism in DM patients, particularly during spring, summer, and autumn.

**Table 4:** The odds ratio of 25-OHD3 metabolites in moderate and serious CKD compared to mild CKD in DM patients

Seasons	25-OHD3			C3-epi-D3 level			%C3-epi-D3		
	OR(95%CI)	Wald *χ*^2^	*P*	OR(95%CI)	Wald *χ*^2^	*P*	OR(95%CI)	Wald *χ*^2^	*P*
All seasons									
Moderate CKD	**0.889 (0.850, 0.929)**	**27.294**	**< 0.001**	**2.434 (1.740, 3.403)**	**27.026**	**< 0.001**	**1.161 (1.083, 1.244)**	**17.777**	**< 0.001**
Serious CKD	**0.894 (0.853, 0.938)**	**21.346**	**< 0.001**	**6.593 (4.786, 9.081)**	**133.231**	**< 0.001**	**1.459 (1.367, 1.556)**	**131.071**	**< 0.001**
Spring									
Moderate CKD	NA	NA	NA	NA	NA	NA	1.040 (0.896, 1.208)	0.267	0.605
Serious CKD	NA	NA	NA	NA	NA	NA	**1.324 (1.153, 1.519)**	**15.881**	**< 0.001**
Summer									
Moderate CKD	NA	NA	NA	NA	NA	NA	**1.348 (1.182, 1.538)**	**19.910**	**< 0.001**
Serious CKD	NA	NA	NA	NA	NA	NA	**1.698 (1.501, 1.922)**	**70.472**	**< 0.001**
Autumn									
Moderate CKD	NA	NA	NA	NA	NA	NA	1.139 (0.997, 1.300)	3.695	0.055
Serious CKD	NA	NA	NA	NA	NA	NA	**1.342 (1.180. 1.525)**	**20.266**	**< 0.001**
Winter									
Moderate CKD	NA	NA	NA	NA	NA	NA	0.865 (0.534, 1.402)	0.346	0.556
Serious CKD	NA	NA	NA	NA	NA	NA	0.753 (0.485, 1.168)	0.205	0.753

Adjusted for gender, and age. “NA”, the variable was excluded from the stepwise analysis due to *P* > 0.10. The results revealed that among 25-OHD3 metabolites, only %C3-epi-D3 was independently associated with moderate and serious CKD severity during specific season when the interaction of the three was considered. Significant values are in bold

**Table 5:** The partial correlation coefficient of 25-OHD3 metabolites with Ca-IP in DM patients

Seasons	25-OHD3			C3-epi-D3 level			%C3-epi-D3		
	*r*_partial_	*t*	*P*	*r*_partial_	*t*	*P*	*r*_partial_	*t*	*P*
All seasons	− 0.047	− 1.135	0.257	− **0.164**	− **4.030**	**< 0.001**	0.030	0.735	0.463
Spring	− 0.079	− 0.856	0.394	− 0.046	− 0.501	0.617	− 0.032	− 0.343	0.733
Summer	− 0.016	− 0.203	0.839	− 0.083	− 1.060	0.291	− 0.060	− 0.773	0.440
Autumn	− 0.027	− 0.344	0.731	− **0.300**	− **4.018**	**< 0.001**	− 0.080	− 1.022	0.308
Winter	− 0.047	− 0.547	0.585	− **0.319**	− **3.896**	**< 0.001**	0.140	1.632	0.105

Adjusted for gender, and age. The results revealed that among 25-OHD3 metabolites, only C3-epi-D3 levels was independently associated with Ca-IP during specific season when the interaction of the three was considered. Significant values are in bold

## Discussion

This study focused on the association of C3-epi-D3 levels and proportions with the metabolic disorders in T2DM patients, and revealed their evident impacts on calcium-phosphorus metabolism and CKD progression during specific season. Specifically, an increased %C3-epi-D3 might serve as a potential risk for moderate CKD severity during summer, as well as for serious CKD severity during spring, summer, and autumn; but C3-epi-D3 levels, rather than %C3-epi-D3, was evidently correlated to calcium-phosphorus metabolism during autumn and winter. However, none of the 25-OHD3 metabolites exhibited evident association with dyslipidemias. Additionally, age and seasonal variations observed in C3-epi-D3 levels, but only seasonal difference observed in %C3-epi-D3.

C3-epi-D3 and 25-OHD3 are isomers for each other, with similar spectroscopic patterns and identical mass and fragmentation patterns [34]. The distinction of C3-epi-D3 to 25-OHD3 lies in the spatial configuration of hydroxyl group at the third carbon atom, which undergoes a transition from α to β. This alteration results in C3-epi-D3 exhibiting minimal or negligible biological activity, significantly inferior to the non-epimer form of 25-OHD [35, 36]. Our study revealed a significant increased C3-epi-D3 level and %C3-epi-D3 as CKD worsened in patients with T2DM. Therefore, due to the inability to identify C3-epi-D3 in spectroscopic or immunoassay detection techniques, an increase in C3-epi-D3 must result in an overestimation of 25(OH)D3 level. This overestimation may not match the development and outcome of T2DM, thus potentially resulting in the previous controversial reports. Furthermore, our study also revealed age and seasonal variations in C3-epi-D3 levels among patients with T2DM, while %C3-epi-D3 exhibited only seasonal differences. Hence, it is crucial to consider age and season factors for properly comprehending the relationship between C3-epi-D3 levels and T2DM. As for %C3-epi-D3, only seasonal stratification analysis maybe must be necessary with emphasis.

Currently, previous studies on the pathological relevance of C3-epi-D3 primarily focus on accurately assessing 25-OHD3 deficiency by distinguishing C3-epi-D3. These studies generally concurred that clinical hazard of patient primarily arise from an insufficiency in 25-OHD3 level [23, 37, 38]. Recent Mendelian randomization reports have suggested the causality between C3-epi-D and some diseases [25, 40], and some clinical and animal investigations have documented the clinical importance of C3-epi-D3 [30, 41]. So, whether an elevated C3-epi-D3 itself has any pathological implications is even more confusing.

It was reported that C3-epi-D3 levels had a property of highly-tracking to 25-OHD3 levels over time [39], suggests a strong correlation between them. However, our study revealed an increase in C3-epi-D3 levels and %C3-epi-D3 accompanied by a decrease in 25-OHD3 levels during CKD progression. These findings deviate from the above property of C3-epi-D3, and strongly suggest that there is a pathological generation mechanism of C3-epi-D3. In contrast, %C3-epi-D3 may provide a more accurate reflection in pathological relevance than C3-epi-D3 levels. C3-epi-D3 levels did not significantly increase in the moderate CKD group. Its association with CKD severity only observed in bivariate Spearman analysis, did not supported by multinomial logistic regression analysis. These disadvantages were speculated to be associated with the property of C3-epi-D3, highly-tracking to 25-OHD3 levels [39]. Finally, our comprehensive seasonal stratified analysis confirmed that %C3-epi-D3, rather than C3-epi-D3 levels, demonstrated an independent association with moderate CKD severity during the summer, as well as serious CKD severity during the spring, summer, and autumn. In conclusion, %C3-epi-D3 is independently associated with the CKD severity in T2DM patients, suggesting that %C3-epi-D3 maybe have pathological value.

Our study also aimed to investigating the potential association between C3-epi-D3 levels and %C3-epi-D3 with dyslipidemias and calcium-phosphorus metabolism. Overall, there was no evident Spearman correlation between C3-epi-D3 levels / %C3-epi-D3 and dyslipidemias (|*ρ*|< 0.3), regardless of seasonal stratification or not. Additionally, before seasonal stratification, no apparent correlation was also observed between C3-epi-D3 levels / %C3-epi-D3 and Ca-IP. However, after considering seasonal stratification, Spearman correlation and partial correlation analysis revealed an evident correlation and an independent association between C3-epi-D3 levels and Ca-IP during autumn and winter, respectively. The Ca-IP reflects the storage of calcium and phosphorus, as well as the balance of calcium-phosphorus metabolism in vivo [42]. Calcium and phosphate homeostasis is tightly regulated by 1,25-dihydroxyvitamin D, which is synthesized from the non-C3-epimer form of 25-OHD in the kidney [43]. It follows that the influence of 25-OHD3 levels on calcium-phosphorus metabolism is more direct than its proportion. Accordingly, C3-epi-D3 levels may have a closer association with calcium-phosphorus metabolism than %C3-epi-D3.

Highlights and limitations: report is limited on the association between C3-epi-D3 generation and metabolic disorders in patients with T2DM. Our study identified an independent association between %C3-epi-D3 and CKD severity in spring, summer, and autumn. However, this finding maybe not generalizable to populations with different diseases or ethnicities. Fortunately, a cohort study also observed an association between C3-epi-D3 and T2DM [25]. Furthermore, our novel finding, an independent correlation between C3-epi-D3 levels and Ca-IP during autumn and winter in T2DM patients, necessitating further validation through more clinical trials, especially comprehensive analysis of 25-OHD2 and 25-OHD3. In addition, this study lacks of regional variation and ethnic diversity, further follow up visit, investigation into other sunshine factors (such as outdoor activities and use of skin creams), so the results may differ over an expanded region, ethnic, or over time.

## Conclusions

This study comprehensively analyzed the differential performance of C3-epi-D3 levels and proportions during specific season in patients with T2DM. Our findings suggest an independent association between %C3-epi-D3 and CKD severity during spring, summer, and autumn, and propose an independent correlation between C3-epi-D3 levels and Ca-IP during autumn and winter. This differential performance indicates their distinct applicability in different metabolic disorders of T2DM patients. Seasonal difference maybe emphasizes the importance of proactive management timing, that is, when do we proactively manage the C3-epi-D3 generation? As for how to manage the C3-epi-D3 generation, it needs further study.

## Supplementary Information

Supplementary Material 1.

## Data Availability

Data is provided within the manuscript or supplementary information files.

## Abbreviations

25-OHD: 25-Hydroxyvitamin D
ADA: American Diabetes Association
C3-epi-D: C3-epimer of 25-hydroxyvitamin D
Ca-IP: Calcium-phosphorus product
CKD: Chronic kidney disease
CysC: Cystatin C
HDL-Chol: High-density lipoprotein cholesterol
HPLC–MS/MS: High-performance liquid chromatography-tandem mass spectrometry
LDL-Chol: Low-density lipoprotein cholesterol
SCr: Serum creatinine
total-Chol: Total cholesterol
T2DM: Type 2 diabetes mellitus
UHPLC-MS/MS: Ultra high-performance liquid chromatography-tandem mass spectrometry
UACR: Urinary albumin/creatinine ratio
