# Supplementary figures and images for: Differential seasonal performance of C3-epi-D3 level and proportion on multiple metabolic disorders in patients with type 2 diabetes mellitus
Source: Eur J Med Res. 2024 Dec 23;29:617. doi: 10.1186/s40001-024-02212-9 (PMC11665098; doi:10.1186/s40001-024-02212-9)

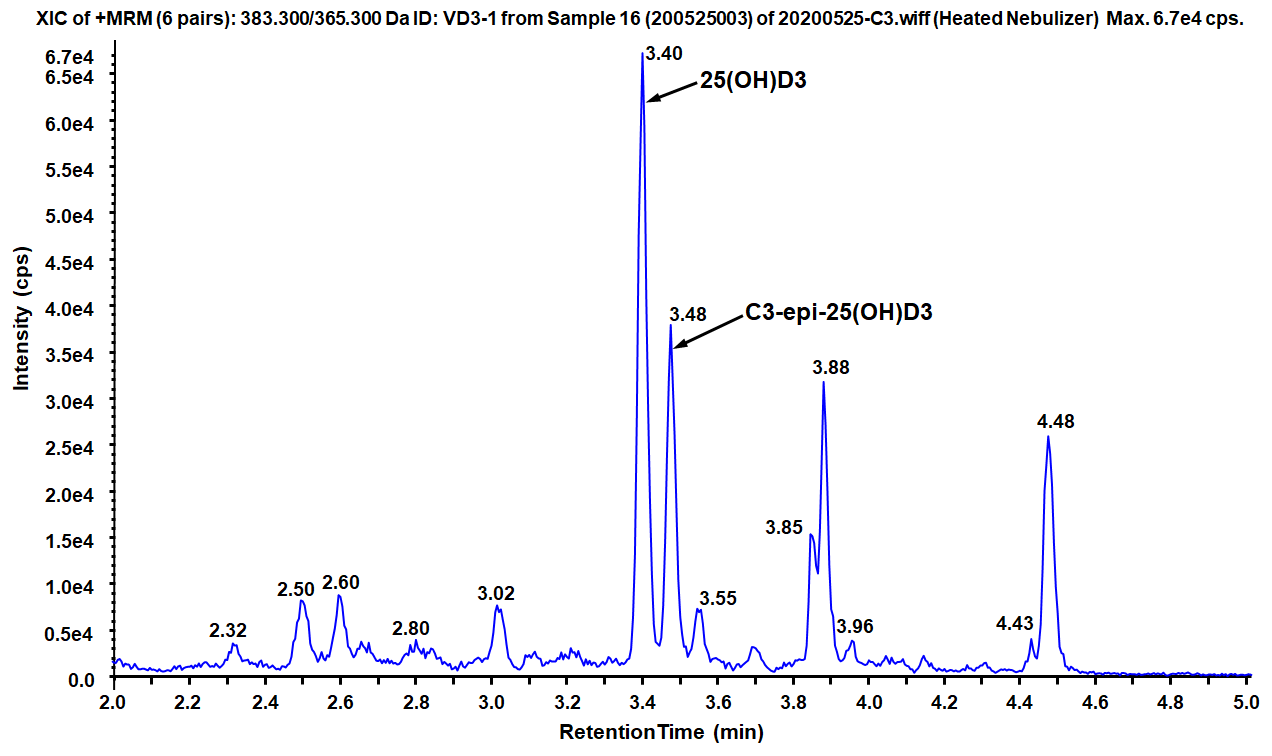

Supplement: Supplementary file 1 — Supplementary Material 1. [file 40001_2024_2212_MOESM1_ESM.tif]
